# Excitability of the Motor Cortex Ipsilateral to the Moving Body Side Depends on Spatio-Temporal Task Complexity and Hemispheric Specialization

**DOI:** 10.1371/journal.pone.0017742

**Published:** 2011-03-09

**Authors:** Femke E. van den Berg, Stephan P. Swinnen, Nicole Wenderoth

**Affiliations:** Motor Control Laboratory, Research Centre for Motor Control and Neuroplasticity, Department of Biomedical Kinesiology, Group Biomedical Sciences, Katholieke Universiteit Leuven, Leuven, Belgium; French National Centre for Scientific Research, France

## Abstract

Unilateral movements are mainly controlled by the contralateral hemisphere, even though the primary motor cortex ipsilateral (M1_ipsi_) to the moving body side can undergo task-related changes of activity as well. Here we used transcranial magnetic stimulation (TMS) to investigate whether representations of the wrist flexor (FCR) and extensor (ECR) in M1_ipsi_ would be modulated when unilateral rhythmical wrist movements were executed in isolation or in the context of a simple or difficult hand-foot coordination pattern, and whether this modulation would differ for the left versus right hemisphere. We found that M1_ipsi_ facilitation of the resting ECR and FCR mirrored the activation of the moving wrist such that facilitation was higher when the homologous muscle was activated during the cyclical movement. We showed that this ipsilateral facilitation increased significantly when the wrist movements were performed in the context of demanding hand-foot coordination tasks whereas foot movements alone influenced the hand representation of M1_ipsi_ only slightly. Our data revealed a clear hemispheric asymmetry such that MEP responses were significantly larger when elicited in the left M1_ipsi_ than in the right. In experiment 2, we tested whether the modulations of M1_ipsi_ facilitation, caused by performing different coordination tasks with the left versus right body sides, could be explained by changes in short intracortical inhibition (SICI). We found that SICI was increasingly reduced for a complex coordination pattern as compared to rest, but only in the right M1_ipsi_. We argue that our results might reflect the stronger involvement of the left versus right hemisphere in performing demanding motor tasks.

## Introduction

Unilateral movements are mainly controlled by the primary motor cortex (M1) of the contralateral hemisphere. However, previous studies have reported that also primary motor cortex ipsilateral (M1_ipsi_) to the moving body side can undergo task-related modulations of activity. Using transcranial magnetic stimulation (TMS) it was shown that performing a forceful, isometric contraction with one hand induced a significant increase of corticomotor excitability in M1_ipsi_, even when the other hand was at rest such that no overt electromyographic (EMG) activity was observed [1]–[8]. Even though facilitation of M1_ipsi_ has been shown repeatedly for strong, isometric contractions, somewhat inconsistent results were obtained during phasic hand or finger movements: Brief, phasic movements requiring only low forces, induced rather inhibition than excitation of M1_ipsi_ [9], [10]. By contrast, rhythmical flexion-extension movements of one wrist increased corticomotor excitability of M1_ipsi_ such that this facilitation mirrored the phasic activity of homologous muscles of the moving hand [11]. Ziemann and Hallett [12] and Tinazzi and Zanette [7] reported increased corticomotor excitability for M1_ipsi_ which was larger when subjects performed complex finger sequences as compared to simple movements.

Functional imaging studies have revealed that the activation of motor areas ipsilateral to the moving hand is asymmetric such that the left hemisphere is more activated when a complex movement task is executed with the ipsilateral, left body side than the right hemisphere during movements with the right body side, or when simple tasks are executed [13]–[17] for a review see [18]. However, these asymmetries were most consistently reported for areas upstream from M1 and, particularly, for parietal and premotor regions, probably because functional imaging offers only limited sensitivity for studying M1. Only a few studies tested hemispheric asymmetries of ipsilateral M1 facilitation using TMS. Stinear et al [6], applied TMS to both hemispheres while the ipsilateral hand performed isometric contractions at different force levels, however, no hemispheric differences were observed. By contrast, Ziemann and Hallett [12] applied TMS to M1_ipsi_ of each hemisphere while right-handed subjects performed a thumb-to-middle-finger opposition task (simple task) versus a sequence of opposition movements from the thumb to index, middle, ring or little finger. They found significant hemispheric asymmetries such that M1_ipsi_ facilitation was larger when the task was executed with the left compared to the right hand and particularly, when subjects had to perform the complex sequencing task.

In the present study we performed two experiments to further investigate behavioural and neural determinants of hemispheric asymmetries in M1_ipsi_ facilitation. In the first experiment we investigated whether ipsilateral facilitation would be modulated when rhythmical wrist movements were executed in isolation or in the context of a simple or difficult hand-foot coordination pattern, and whether this modulation would differ for the left versus right hemisphere. It has been shown previously that task complexity of these multilimb coordination tasks depends on the spatiotemporal pattern between hand and foot movements such that coordination control is easier when both limbs move into the same direction (in-phase) than when limbs move into opposite directions (anti-phase)[19]–[21]. Importantly, using this paradigm, subjects perform identical wrist movements across all conditions, such that the output of the investigated muscles can be kept constant while task complexity is systematically varied.

In the second experiment we tested whether hemispheric asymmetries in the modulation of M1_ipsi_ excitability due to coordinative task complexity might result from reduced intracortical inhibition. Previous studies indicated that the M1_ipsi_ facilitation emerges, at least partly, at the cortical level: First, Tinazzi and Zanette [7] reported increased M1_ipsi_ excitability only for TMS which activates corticospinal neurons transsynaptically but not for transcranial electric stimulation (TES) that activates corticospinal axons directly, probably within white matter structures. Second, it was shown that M1_ipsi_ is facilitated while responses to cervicomedullary stimulation of the descending tracts were unchanged [1], [11]. Third, paired-pulse short-interval intracortical inhibition (SICI), which is mediated by GABAergic, cortical interneurons [22] was decreased due to forceful isometric contractions of the opposite hand [2] and this decrease became stronger the more force was applied [3]. Moreover, at high force levels, the decrease of SICI was correlated to an increase in interhemispheric inhibition suggesting that intracortical and transcallosal pathways interact to control M1_ipsi_ [3]. However, it is currently unknown whether SICI of the ipsilateral M1 also changes during phasic movements and whether these potential modulations depend on the complexity of the task and/or which body side performs the task.

## Methods

### 2.1 Subjects

Twelve healthy volunteers (age 20–23 yrs, 12 female) participated in *experiment 1* and eight subjects (age 19–24 yrs, 3 female) in *experiment 2*. They were all right-handed, as assessed by the Edinburgh Handedness Inventory [23] and naïve to the task. Subjects were screened for contra-indications for TMS such as epilepsy, migraine, implants in the head as well as for overt sensorimotor and other major physical deficits. The study was approved by the Medical Ethics committee of the University Hospital at the K.U. Leuven in accordance to the Declaration of Helsinki (1964) and each subject read and signed a written informed consent prior to the experiment.

### 2.2 General setup

**Figure 1:**
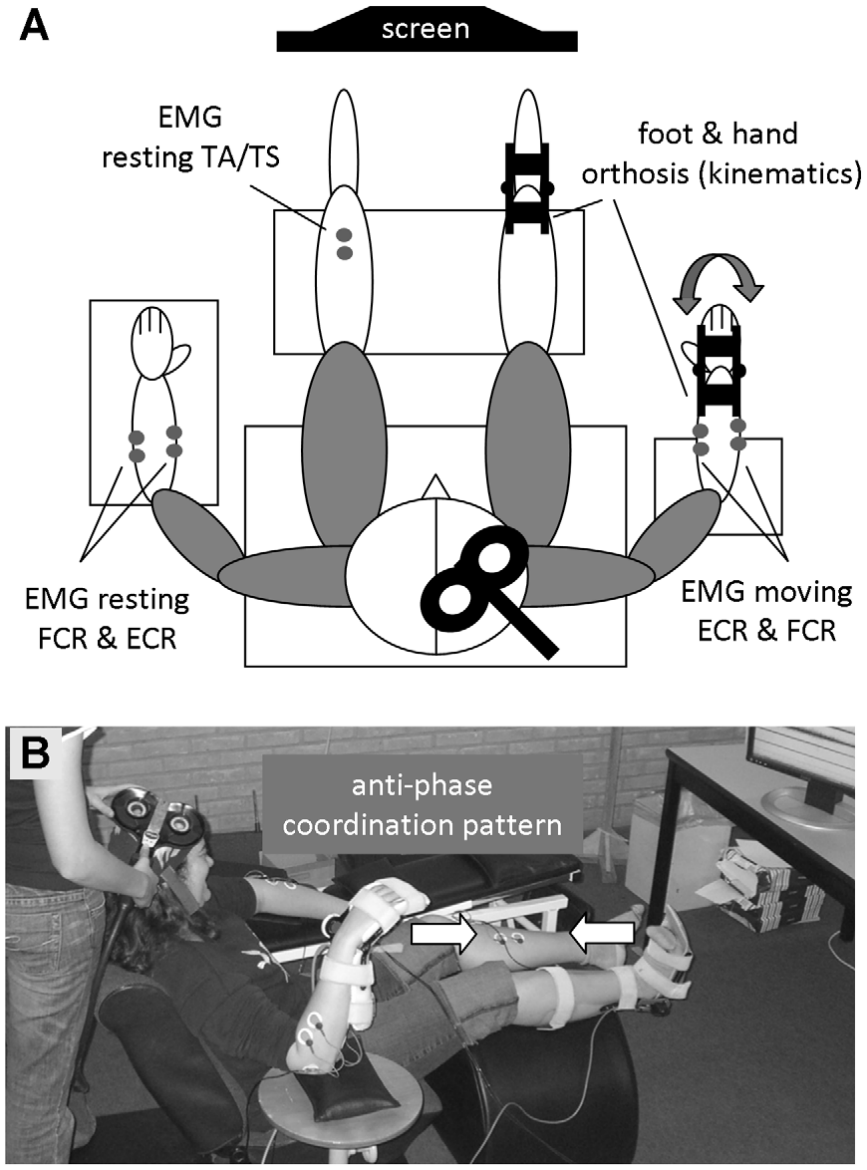
Experimental setup (A) and illustration of performance of the anti-phase coordination pattern (B). Abbreviations: flexor carpi radialis (FCR); extensor carpi radialis (ECR); tibialis anterior (TA); triceps Surae (TS). Consent to publication was obtained from the participant shown at the photograph.

Measurements were performed while either the right arm and/or leg were active whereas the left arm and leg were resting, or vice versa. Subjects were comfortably seated in a low chair with their legs outstretched on a soft support. The resting arm and leg were fully supported such that subjects could completely relax. The leg of the actively moving foot was positioned such that the calf was supported but the ankle could move without restrictions. The elbow of the actively moving arm was supported such that the forearm was held upright and the wrist could move freely (figure 1). Subjects were instructed to fully extend their wrist, to ensure that both the wrist flexor and extensor had to be activated to move the wrist against gravity in the respective part of the movement cycle. Displacement data of the moving hand and foot were measured by shaft encoders (HP Hewlett-Packard, Malaysia) mounted to custom made wrist and ankle orthoses. The axis of each orthosis was aligned to the axis of rotation of the wrist/ankle joint and flexion and movements were measured with a frequency of 100 Hz and a spatial resolution of 0.18°.

**Figure 2:**
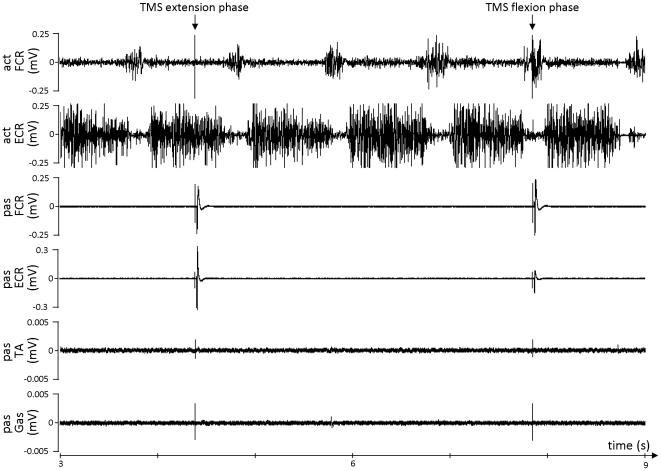
Typical example of the EMG signals registered from the moving wrist muscles (upper two panels), the resting wrist muscles (middle two panels) and the resting foot muscles (lower two panels) during rhythmical hand flexion and extension movements. Arrows indicate TMS stimulation timed such that the first one was positioned within the extension and the second within the flexion burst. TMS was applied in the hemisphere ipsilateral to the moving hand and evoked clear MEPs in the contralateral resting FCR and ECR. Abbreviations are identical to figure 1.

The electromygraphic (EMG) activity of the Tibialis Anterior (TA) and Triceps Surae (TS) of the resting leg and the Extensor Carpi Radialis (ECR) and Flexor Carpi Radialis (FCR) of both arms were recorded throughout the experiment (Mespec 8000, Mega Electronics Ltd., Kuopio, Finland) with two disposable Ag–AgCl surface electrodes (Blue Sensor P-00-S, Ambu, Ølstykke, Denmark) placed over the muscle belly and one reference electrode placed over a bony structure. Each EMG channel was measured with a frequency of 5 kHz, amplified, filtered (30–1500 Hz) and displayed on a computer screen in front of the subject (CED Power 1401, Cambridge Electronic Design, Cambridge, UK and Signal 3.03 software). Using this online EMG feedback, subjects were trained to fully relax their resting limbs even when movements were performed with the other body side (figure 2). Also during the experiment, EMG activity of the resting limbs was closely monitored by the subjects and the experimenters and trials were repeated when overt EMG activity was observed. Due to this setup they had only partial vision of their limb movements and only in the periphery of their field of view. Thus, the movement tasks were mainly executed under proprioceptive control.

### 2.3 Experiment 1

#### 2.3.1 Task

The participants were instructed to execute five different experimental conditions: (1) rhythmical plantarflexion and dorsiflexion movements with the foot (called “foot flexion” and “foot extension”, respectively, in the remainder of the manuscript), (2) rhythmical flexion and extension movements with the hand while the foot was resting, (3) rhythmical in-phase coordination, i.e. hand and foot were simultaneously flexed and extended, (4) rhythmical anti-phase coordination, i.e. the hand was extended when the foot was flexed and vice versa, and (5) a rest condition. All movements were produced rhythmically as paced by an auditory metronome at 1 Hz. Each trial lasted 20 seconds and started with the wrist and ankle in neutral position. Subjects were instructed to synchronize their movements to the metronome such that the wrist was flexed on the beat in the hand (HAND), in-phase (IN) and anti-phase condition (ANTI). When only the foot was moved (FOOT condition), ankle flexion was performed on the beat. Additionally, there was a rest condition in which subjects did not move, but remained completely relaxed while the metronome produced a 1 Hz rhythm. For each condition, several training trials were performed prior to the TMS measurements to practice the different tasks while relaxing the non-involved body side. During the experiment, each of the movement conditions was performed 8 times and rest 4 times in pseudo-random order. The experiment consisted of two separate sessions that took place at two different days. Half of the subjects performed all tasks first with their left and then with their right body side while the order was reversed for the other half of the subjects.

#### 2.3.2 TMS-procedure

Single-pulse transcranial magnetic stimulation (TMS) was delivered through a figure-of-eight shaped stimulation coil (mean diameter of each wing, 70mm) connected to a Magstim 200 (Magstim Company Ltd., Carmarthenshire, UK) and motor evoked potentials (MEPs) were recorded in the contralateral, resting wrist muscles. The subject wore a tight-fitting cotton cap with a 1-cm grid. The coil was placed tangentially to the scalp over the primary motor cortex with the handle pointing backward and 45° away from the midline. The stimulator produced a near monophasic wave form and, with this coil orientation, the induced current was directed from posterior-lateral to anterior-medial which activates corticospinal neurons predominantly via horizontal corticocortical connections [24].

The hotspot of the FCR, i.e. the optimal position to elicit maximal MEPs in the contralateral limb, was determined and marked on the swimming cap. The rest motor threshold (RMT) was determined as the stimulation intensity that elicited a MEP peak-to-peak amplitude >0.05 mV in the relaxed FCR in at least five out of ten consecutive stimuli [25]. Even though the parameter setting procedures focused on the FCR, ECR parameters were assumed to be satisfactorily similar, due to the overlapping representations of forearm flexors and extensors [26]. During the experiment, the stimulation intensity was set at 125% of the FCR RMT and the coil was placed over the primary motor cortex ipsilateral to the moving body side (M1_ipsi_) to record MEPs in the contralateral, resting muscle.

The participants performed 36 trials in total and four stimulations (on average 5s apart) were applied during each trial. The absolute timing of the stimulation was randomized, but the pulses were applied either 150ms before the beat of the metronome (i.e. targeting the flexion burst of the moving hand) or 400 ms after (i.e. targeting the extension burst). Each movement condition was repeated 8 times (2 stimulations during extension burst, 2 during flexion burst per trial) and the rest condition was repeated 4 times (4 stimulations during rest). Thus, there were 16 stimulations for each condition and each flexion/extension phase.

#### 2.3.3 Data-analysis of EMG and kinematics of the moving limbs

For the hand-foot coordination conditions, the relative phase angle between limbs was calculated from the displacement data of hand and foot by: 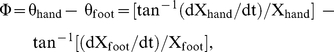



where θ_hand_ is the phase of the hand movement at each sample; X_hand_ is the position of the hand after rescaling to the interval [−1,1] for each movement cycle, and dX_hand_/dt is the normalized instantaneous velocity. The mean continuous relative phase and its standard deviation was determined for each trial and the absolute phase error was calculated as the absolute difference between the mean relative phase and the target phase (i.e 0 deg for the IN and 180 deg for the ANTI condition). Additionally we determined the mean movement amplitude and cycle duration for each limb and condition. For the ECR and FCR of the actively moving hand, EMG was determined as the root-mean-square (RMS) value of the EMG signal during the last 50 ms prior to stimulation.

#### 2.3.4 Data-analysis of corticomotor excitability in M1 ipsilateral to the moving limbs

Corticomotor excitability of the resting FCR and ECR, i.e. elicited via the hemisphere ipsilateral to the moving limbs, was determined by the peak-to-peak amplitude of the MEPs. All EMG traces were visually inspected and MEPs were removed from subsequent analysis (9.2% in total) (a) when overt EMG activity emerged in the resting hand or foot muscles 50 ms prior to stimulation, (b) when the stimulation fell outside the targeted EMG burst of the active, homologous muscle or (c) when subjects performed the wrong coordination pattern. MEP-amplitudes of the FCR and ECR were averaged within each subject such that one mean value was calculated for each muscle, condition and phase (only for the movement conditions). Finally, the MEP-amplitudes were normalized relative to the rest MEPs (MEP_norm_ = MEP_movement condition_/MEP_rest_).

#### 2.3.5 Statistical analysis

All statistical analyses were performed with Statistica 8.0 (StatSoft, Inc., Tulsa, USA). Differences between RMT of the left versus right hemisphere were tested by dependent t-tests. The absolute phase error and the standard deviation of the relative phase were subjected to an analyses of variance for repeated measurements (repeated measures ANOVA) with the within factors *moving body side* (left, right) and *coordination pattern* (IN, ANTI). Movement amplitude and cycle duration were analyzed by a repeated measures ANOVA with the factors *moving body side* (left, right), *condition* (single limb, IN, ANTI) and *limb* (hand, foot). The active EMG of the moving limbs as well as the normalized MEP amplitudes generated from the hemisphere ipsilateral to the moving body side were subjected to a repeated measures ANOVA with the factors *hemisphere* (left, right), *condition* (HAND, FOOT, IN, ANTI), *muscle* (FCR, ECR) and *contraction phase* of the homologous, contralateral muscle (active, passive). Significant effects were further tested with Fisher LSD posthoc tests. The criterion for statistical significance was α = 0.05. Descriptive statistics will be reported as mean and standard error in text and figures.

### 2.4 Experiment 2

#### 2.4.1. Task

The task was identical to experiment 1, however subjects performed only 3 different conditions: HAND, ANTI and REST, each lasting 12 s. Prior to testing, subjects familiarized themselves with the task and practiced the coordination pattern while the resting body side was completely relaxed. In the main experiment each of the REST, HAND and ANTI conditions was tested 14 times, in a randomised order and, per trial, two TMS stimulations were applied that were at least 4 s apart.

#### 2.4.2 TMS procedure

TMS was applied over M1_ipsi_ with a 70 mm figure eight coil connected to a Magstim 200 stimulator through the BiStim Module (Magstim, Whitland, Dyfed UK). After the hotspot of the ECR was located, RMT and the active motor threshold (AMT) were determined, defined as the minimal stimulus intensity necessary to produce MEPs larger than 0.1 mV in at least five out of ten consecutive trials while subjects maintained a slight voluntary contraction of the ECR at approx. 3% of the maximal voluntary contraction.

SICI was measured by a double-pulse paradigm such that in half of the trials a superthreshold test stimulus (TS) was preceded by a subthreshold conditioning stimulus (CS) [22]. The interstimulus interval was set to 2.5 ms, the optimal interval to induce intracortical inhibition [27]–[29]. As experiment 1 has shown that excitability of M1_ipsi_ differs depending on the task and hemisphere tested, we attempted to adjust the test stimulus (TS) intensities such that MEP responses with an amplitude of 0.7–1.0 mV were evoked by single pulse stimulation for all conditions. The conditioning stimulus (CS) intensity was set such that MEP amplitude was reduced by approximately 50% when the TS was preceded by the CS at REST. Intensities were adjusted in the beginning of each session and kept constant for the remainder of the experiment.

In each trial one single (TS) and one double pulse stimulation (CS+TS) were delivered. In the REST condition, this occurred at random time points and with an interval between single and double pulse stimulation of at least 4 seconds. In the HAND and ANTI condition the EMG burst of the moving ECR was detected online and used to trigger the stimulation over M1_ipsi_. Stimulation was provided during the burst because experiment 1 showed M1_ipsi_ facilitation to be larger when the homologous muscle of the other body side is active. The interval between the first and second stimulation was at least 4 seconds. A trial was repeated (a) if the experimenters observed overt EMG activity in the resting body side, (b) if only one stimulation pulse was given, or (c) in case the subject did not perform the coordination task correctly.

#### 2.4.3 Data-analysis

EMG and kinematics of the actively moving body side were analyzed analogous to experiment 1. For the TMS data collected from M1_ipsi_, MEP amplitudes were determined as described above and the percentage of intracortical inhibition was calculated by %SICI = (MEP_TS_-MEP_CS+TS_)/MEP_TS_*100 (note that large values indicate a high level of inhibition).

#### 2.4.4 Statistics

Differences between hemispheres for RMT, AMT, CS and the coordination performance of ANTI were analysed by dependent t-tests. Active EMG of the ECR of the moving hand was analysed by a repeated measures ANOVA with the factors *moving body side* (left, right), *condition* (HAND, ANTI) and *stimulation* (TS, CS+TS). Finally, TS intensities, MEP amplitudes and %SICI were analyzed by a repeated measures ANOVA with the factors *moving body side* (left, right), *condition* (REST, HAND, ANTI) and gender (male, female) as a covariate of no interest.

## Results

### 3.1 Experiment 1

RMT was similar between the hemispheres and ranged from 38–55% of the maximum stimulator output (average of 46±1.6%) for the left hemisphere and from 36–59%, (average of 45.6±1.8%) for the right hemisphere (p>0.05).

#### 3.1.1 Movement performance of the active body side

Subjects complied well with the required cycling frequency for all conditions even though small but significant differences were found such that movements were slightly faster for ANTI (0.980±0.005 s) than IN or the single limb conditions (both 0.983±0.003 s) and also when moving with the right body side (0.980±0.004 s) as compared with the left (0.984±0.004 s) (F(2, 22)≥4.4184, p<0.01). Movement amplitudes were generally larger for hand (115±23 deg) than foot movements (41±11 deg) (F(1,11) = 74, p<0.0001). The ANOVA revealed also a significant *condition* x *limb* interaction (F(2,22) = 6.4401, p<0.01) which was driven by a significantly larger hand amplitude for HAND only (123±15 deg) than for IN (111±14 deg) and ANTI (109±13 deg), while the foot amplitude remained virtually unchanged.

The absolute relative phase error did not differ between IN and ANTI coordination or between the body sides (overall 16±1.1 deg; F(1,11) = 2.0609, p = 0.18). However, the standard deviation of the relative phase was slightly higher for ANTI (26.0±1.3 deg) than for IN (24.3±2.1 deg) and when movements were performed with the left body side (26.3±1.4 deg) than with the right (24.0±2.1) [F(1,11) ≥5.5, p = 0.03 and p = 0.04, respectively].

#### 3.1.2 EMG of the actively moving hand

**Figure 3:**
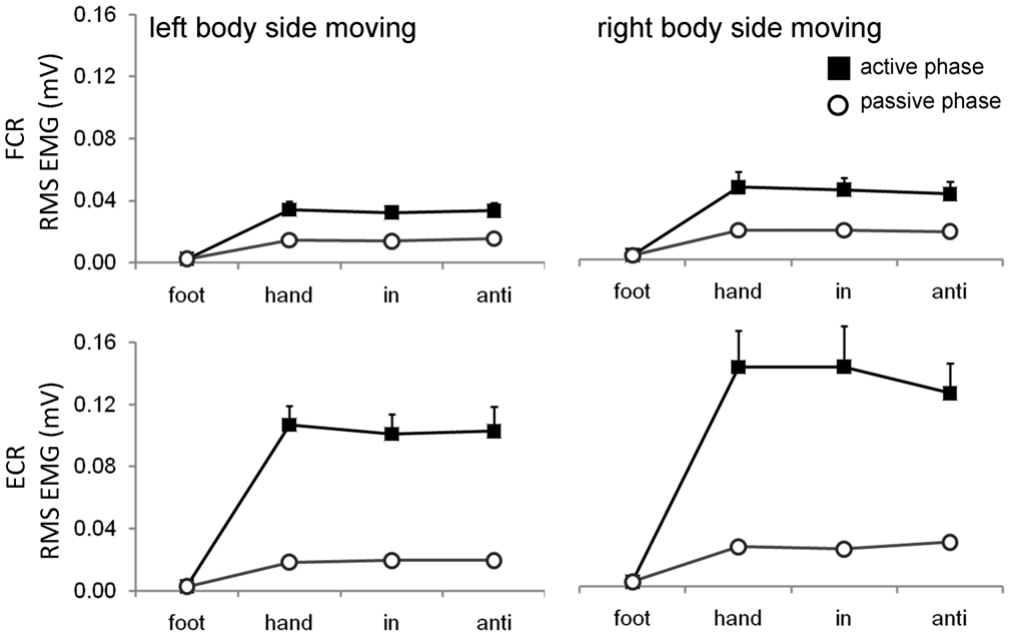
Mean EMG activity of the ECR and FCR of the actively moving hand during TMS in experiment 1. Data are shown for all movement conditions executed with the left or right body side and for the active phase (square) and passive phase (circle) of each wrist muscle.

Only for the HAND, IN and ANTI conditions, EMG activity of the wrist muscles was substantially larger during the active than the passive phase (figure 3). This indicates that subjects complied well with the imposed timing and that our stimulation fell reliably within the burst of the ECR when TMS was applied 400 ms after the beep of the metronome and the burst of the FCR when stimulation was applied 150 ms before the beep of the metronome. During the active phase, EMG activity was significantly higher for the ECR (which had to move the hand during most of the extension phase against gravity) than for the FCR (which was activated only during the beginning of the flexion phase as anti-gravitational muscle) (muscle × condition × phase interaction F(3,33) = 38.512, p<0.00001). Muscle activity was slightly stronger when the right than when the left body side was moved (hemisphere × condition interaction F(3,33) = 3.0129, p = 0.04387).

#### 3.1.3 Corticospinal excitability of M1_ipsi_ as indicated by the normalized MEP-amplitude

**Figure 4:**
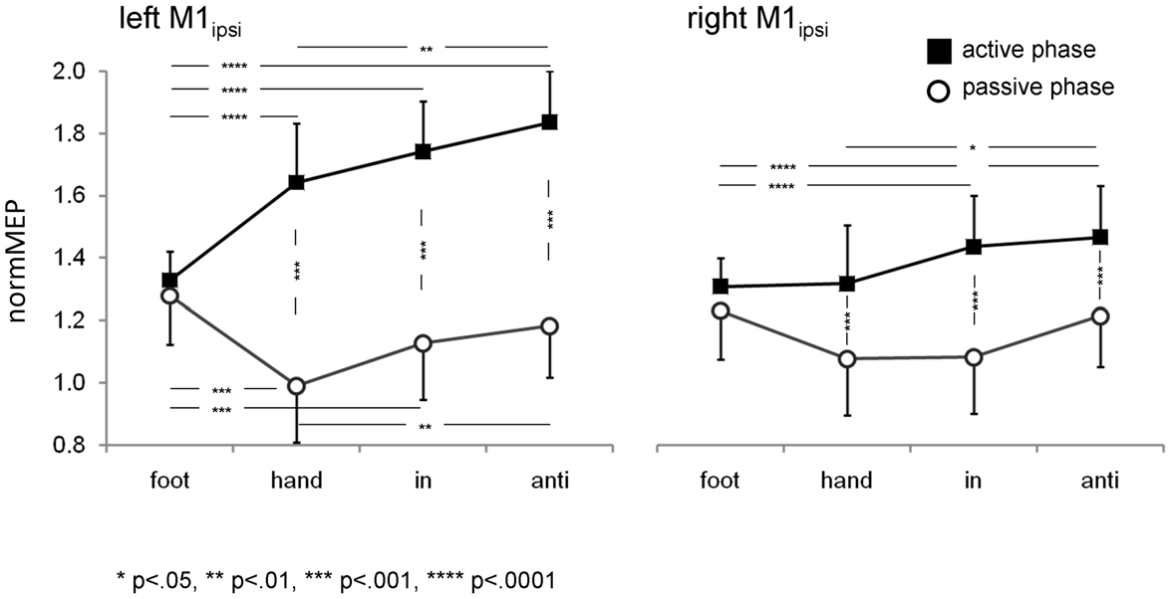
Mean normalized amplitudes of MEPs evoked over the left or right primary motor cortex ipsilateral to the moving body side in experiment 1. Data are shown for all movement conditions and when the homologous muscle of the moving hand was either active (squares) or passive (circles). Significant differences between conditions are indicated by * (p<0.05), ** (p<0.01), *** (p<0.001), **** (p<0.0001).

Performing movements with one body side had a significant influence on the corticospinal excitability of M1_ipsi_. Statistics of the normalized MEP amplitudes revealed a significant effect of *condition* (F(3,33) = 3.9105, p<0.05), *contraction phase* (F(1,11) = 16.365, p<0.005), *hemisphere* x *contraction phase* (F(1,11) = 5.8727, p<0.05) and *condition* × *contraction phase* (F(3, 33) = 11.216, p<0.00005). However, these effects can best be understood in light of the significant *hemisphere* × *condition* × *contraction phase* interaction (F(3,33) = 4.4635, p<0.01) shown in figure 4. For all conditions involving hand movements (i.e. HAND, IN, ANTI), MEP amplitudes of M1_ipsi_ were significantly larger when the contralateral homologous muscle was in the active phase than in the passive (p<0.0001). Importantly, during the active phase, there was a substantial hemispheric asymmetry such that M1_ipsi_ responses were significantly larger in the left than in the right hemisphere for HAND, IN and ANTI (p<0.0001).

In the left hemisphere (figure 4, left panel), corticomotor excitability of M1_ipsi_ during the active phase was lowest in the FOOT condition, which differed significantly from all other conditions (p<0.0001). NormMEP was highest for the ANTI condition and differed significantly from HAND (p<0.01). Similar, albeit smaller modulations for the active phase were found in the right hemisphere (figure 4, right panel) where normMEP was smallest for FOOT (which differed significantly from IN and ANTI, p<0.01) and largest for ANTI that differed also significantly from HAND (p = 0.03).

Finally, we performed an extra analysis on the data during the active phase only to test whether hemispheric asymmetries of M1_ipsi_ facilitation (i.e. higher responses for the left than the right hemisphere) would be larger for the difficult ANTI than the easy HAND condition. However, statistics revealed no significant interaction effect (F(2,22) = 0.28303, p = 0.75620) indicating the hemispheric asymmetries in ipsilateral facilitation were similar across all tasks involving hand movement.

In summary, M1_ipsi_ was mirrored the activity of the moving wrist muscles, such that facilitation was stronger when the stimulation fell into the active phase of the homologous muscle. This effect was more pronounced in the left than in the right M1_ipsi_. Additionally, facilitation was modulated by coordination complexity, being significantly stronger when subjects performed a complex hand-foot coordination pattern (ANTI) as compared to simple hand movements. In the next experiment, we specifically tested whether consistent effects would be observed for intracortical inhibition in M1_ipsi_.

## Results

### 3.2 Experiment 2

RMT and AMT were similar and not significantly different between hemispheres (RMT_left_ = 42.9±1.7%, RMT_right_ = 45.1±1.6%; AMT_left_ = 38.4±1.9%; AMT_right_ = 39.1±1.7% of maximal stimulator output) (p≥0.17). Also CS intensity expressed as %AMT was largely comparable between hemispheres (CS_left_ = 86.2±5.1, CS_right_ = 94.8±3.0) (p = 0.09).

#### 3.2.1 Movement pattern of the active body side and EMG of the actively moving hand

Subjects complied well with the required movement frequency and there were no significant differences between the HAND and ANTI condition or between hemispheres (overall movement frequency = 1.03±.01 Hz, p≥0.19). Movement amplitude was slightly larger for HAND (67±9 deg) than for ANTI (62±9 deg) (F(1,7) = 42.4; p<0.001) but did not differ between moving with the left versus right body side (p≥0.68). For ANTI the overall relative phase error was 9.6±1.5 deg and the relative phase variability was 30.6±4.2 deg but there were no significant differences between hemispheres (p≥0.09). Mean active burst EMG of the ECR was similar for HAND (0.197±0.11 mV) and ANTI (0.189±0.1 mV) (F(1,6) = 2.5; p≥0.16).

#### 3.2.2 Corticospinal excitability and SICI of M1_ipsi_

**Figure 5:**
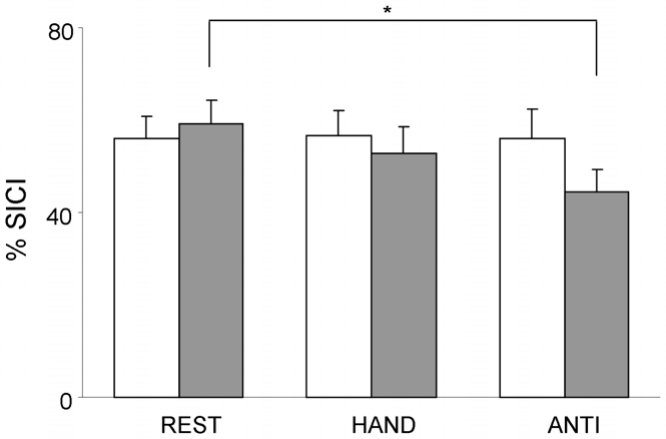
Intracortical inhibition in the left (open bars) and right primary motor cortex (grey bars) ipsilateral to the moving body side is shown for all conditions of experiment 2. Significant differences between conditions are indicated by * (p<0.05).

We aimed to adjust TS intensities such that MEP_TS_ amplitudes were matched across all conditions, ranging between 0.7–1 mV. However, post-hoc analyses revealed that the MEP_TS_ amplitude evoked in left M1_ipsi_ during REST was significantly smaller than for all other conditions, which were well matched (table 1) (main effect in condition: F(2,12) = 4.6 p<0.05; hemisphere × condition: F(2,12) = 4.3; p<0.05). %SICI varied across tasks and hemispheres (figure 5). When stimulating the left hemisphere, intracortical inhibition remained relatively constant across conditions. By contrast, for the right hemisphere, inhibition was increasingly released such that %SICI was highest at REST and lowest for ANTI. Statistics confirmed this differential behavior by means of a significant interaction between hemisphere and condition (F(2,12) = 4.5 p<0.05). Post hoc analyses indicated a significant difference between the REST and ANTI condition in the right hemisphere (p<0.05), whereas, the difference between HAND and ANTI just failed to reach significance (p = 0.063).

**Table 1:** MEP peak-to-peak amplitude measured in experiment 2 in response to the test stimulus (TS).

	Left MEP_TS_ (mV)	Right MEP_TS_ (mV)
Rest	.60*±.21	.93±.33
Hand	.97±.44	.99±.33
Anti	.93±.31	1.02±.30

* indicates that the MEP amplitude evoked via the left hemisphere during rest was significantly smaller than for the other condition (p<0.05).

## Discussion

Here we measured corticomotor excitability of the primary motor cortex ipsilateral to the moving body side when subjects performed rhythmical flexion and extension movements of the wrist either in isolation or as part of a simple (in-phase) or more demanding hand-foot coordination pattern (anti-phase). As a novel result we showed that ipsilateral facilitation of wrist representations in M1 increased significantly when the wrist movements were performed in the context of a demanding hand-foot coordination task. Our data revealed a clear hemispheric asymmetry such that MEP responses were significantly larger when elicited from the left M1_ipsi_ than from the right. Moreover, we found that intracortical inhibition as quantified by SICI was reduced in right but not left M1_ipsi_.

### Corticomotor excitability of M1_ipsi_ is higher when homologous muscles of the other body side are moved

In agreement with previous studies [11], corticomotor excitability of M1_ipsi_ changed substantially as a function of the muscular activity of the homologous muscles of the moving body side. This finding was further supported by the FOOT condition that influenced the excitability of wrist muscles in M1_ipsi_, however, this effect was significantly smaller than when homologous wrist muscles were activated, particularly, during the coordination tasks. The finding that ipsilateral facilitation is strongest in homologous muscles is also interesting in the context of interlimb coordination. It has been shown that rhythmical movements are tightly coupled when they are performed with the same effectors of both body sides (i.e. hand_left_-hand_right_ or foot_left_-foot_right_ coordination) [20], [30], [31] or with different effectors of the same body side (i.e. hand_left_-foot_left_ or hand_right_-foot_right_ movements) [32]–[34]. By contrast, motor actions can be performed with remarkable independence when the wrist of one body side is moved together with the foot of the other body side (i.e. hand_left_-foot_right_ or hand_right_-foot_left_). Our finding that M1_ipsi_ facilitation was strongest when the homologous hand muscle was activated as compared to a non-homologous hand or foot muscle is very much in line with the behavioural results and suggests that M1_ipsi_ facilitation and interlimb coordination might reflect the same physiological phenomenon and probably arise via callosal pathways that are slightly denser between homologous than non-homologous motor areas [35].

### Corticomotor excitability of M1_ipsi_ depends on movement complexity and is stronger in the left than in the right hemisphere

Subjects had to move their wrist in isolation or together with foot movements either in accordance to the simple IN or more complex ANTI pattern. Importantly, wrist movements during ANTI were performed with the same speed and the same or slightly smaller movement amplitude than during HAND/IN and also the active EMG of the wrist muscles did not differ between movement conditions and was only slightly larger when the right than when the left body side performed the task. Thus, hand movements were largely similar across conditions and moving body side such that only the demanding coordination context could have induced the high facilitation of M1_ipsi_ during the ANTI task. This notion is also in line with functional imaging studies indicating that hand-foot coordination according to the ANTI pattern activates areas upstream from M1 more strongly than the IN pattern [36]–[38]. Our results extend previous findings of Ziemann and Hallett [12] who reported a similar increase of ipsilateral facilitation when subjects performed complex compared to simple finger sequences. However, the advantage of our paradigm is that movement characteristics of the investigated muscles were kept constant while task complexity was varied in a systematic way.

In line with Ziemann and Hallett [12] we found that M1_ipsi_ facilitation was stronger in the left than the right hemisphere. Importantly, subjects were able to perform the motor tasks nearly equally well with their left and right body side as there were no significant differences in mean coordination performance between hemispheres (as measured by the relative phase error) and only minor differences in coordination stability indicating that ANTI was somewhat harder to control than IN and that the left body side moved less consistently than the right. Moreover, the stronger facilitation of left M1_ipsi_ compared to right M1_ipsi_ was found to an equal extent for HAND, IN, or ANTI movements. These results indicate that task complexity modulated the extent of ipsilateral facilitation, but not the extent of hemispheric asymmetries.

### Hemispheric asymmetries of SICI

In experiment 2, we measured changes of intracortical inhibition of the ECR in M1_ipsi_ while the homologous muscle was either at REST or was activated during HAND and ANTI movements. We found that %SICI decreased significantly with complexity but only for right M1_ipsi_ and not for left M1_ipsi_. The right hemisphere result is in line with Muellbacher et al [2], who only tested the right hemisphere and found that voluntary activation of the right APB decreased SICI in the right M1_ipsi_. Similarly, Perez et al [3] measured SICI in the right hemisphere while subjects performed isometric contractions at increasing force levels with their right hand. SICI varied in a task specific way and was increasingly released and significantly lower for a strong isometric contraction at 70% as compared to 10%. Our data extend these results, by indicating that %SICI of right M1_ipsi_ tended to change parametrically when subjects performed hand movements either in isolation or in the context of a demanding coordination task.

It is important to note that the produced wrist movements were well matched with respect to movement frequency, movement amplitude, EMG activity of the moving ECR as well as relative phase error and variability for ANTI, that did not differ between hemispheres. We experienced difficulties in matching MEP_TS_ amplitudes across conditions, however, recent experiments have indicated that this has no influence on the results when SICI is expressed relative to the unconditioned MEP amplitude (i.e. %SICI as reported in our present study) [39], [40]. In our study, %SICI was very similar and not significantly different for the REST condition of the left versus right hemisphere. Thus, it is justified to argue that the differential modulation of %SICI across conditions and in the right versus left hemispheres can not be explained by sub-optimally matched MEP_TS_ amplitudes. Instead, our data suggest that also intracortical inhibition of M1_ipsi_ differs between the right versus left hemisphere.

### Potential mechanisms underlying hemispheric asymmetries of M1_ipsi_ facilitation and %SICI

Even though facilitation of the hemisphere ipsilateral to a moving limb has been demonstrated repeatedly and with different methods [12], [17], [41], [42] for a review see Serrien et al [18], it is not completely clear which mechanisms or anatomical pathways cause this effect and why ipsilateral activity is usually larger in the left than the right hemisphere when tested in right-handed subjects. There are three potential levels of the nervous system (which are not mutually exclusive) that might contribute to M1_ipsi_ facilitation and/or to hemispheric asymmetries of this effect: 1) spinal cord physiology, 2) M1-M1 interactions via transcallosal pathways or 3) functional asymmetries in M1 or areas upstream from M1.

First, it has been shown that strong isometric contractions or rhythmical movements of one hand lead to a depression [1], [11] and an additional rhythmic modulation [11] of H-reflexes of the resting hand. This suggests that movements with one hand might modulate segmental inputs to spinal motorneurons controlling the contralateral hand, probably via (presynaptic) inhibition of Ia afferents [1], [11]. However, the same studies have shown that M1_ipsi_ was facilitated while responses to cervicomedullary stimulation of the descending tracts were unchanged, indicating that excitability of the spinal motorneuron pool was not affected by movements of the opposite limb [1], [11]. Thus, even though a spinal contribution can not be ruled out completely, it appears that the facilitation of responses from M1 ipsilateral to a moving limb emerges to a large part at the cortical level. Moreover, previous studies comparing H-reflexes between the left and right body side did not find asymmetries in healthy subjects [43], making it unlikely that the strong left-right differences of M1_ipsi_ facilitation found in our study emerged at the spinal level.

Second, at the cortical level it has been shown that corticomotor excitability is strongly influenced by inhibitory and facilitatory circuits that act either locally within M1 or via transcallosal M1-M1 projections. Results from Perez et al [3] and our own findings indicate that SICI was reduced in the right hemisphere which can explain the increased corticomotor excitability of right M1_ipsi_. Interestingly, Perez at al [3] have also shown that for strong isometric contractions with the right hand, interhemispheric inhibition exerted from the contralateral left to the ipsilateral right hemisphere interacted with SICI in M1_ipsi_, such that SICI was weak when IHI was strong and vice versa. Applied to our data, this would suggest that interhemispheric inhibition might have been asymmetric between hemispheres, being larger from left to right than vice versa. This is generally consistent with the view that, during motor task preparation and/or execution the left, motor-dominant hemisphere (in right handed subjects) has a stronger influence on the right, motor non-dominant hemisphere than vice versa [18], [44]. It is possible that this asymmetry reflects structural features of the corpus callosum [45]. However, studies measuring IHI which is related to the structural integrity of the corpus callosum [46], revealed inconsistent results: Some experiments showed that IHI measured at rest was stronger from the dominant to the non-dominant hemisphere [47]–[49], while others revealed no asymmetries [50], [51]. Also when IHI was correlated with brain activity in M1_ipsi_ either a positive [42], negative [52] or no correlation [13] was found. Thus, more research is needed to establish a convincing link between corpus callosum structure and hemispheric asymmetries in M1_ipsi_ facilitation.

Alternatively, it is possible that there are hemispheric asymmetries concerning the function of M1 or upstream motor areas. There is ample evidence that, in right handed subjects, the left hemisphere is involved in the control of complex motor tasks performed with either body side (for a review see [18]). This asymmetry is particularly pronounced for parietal and premotor areas that are believed to contain “movement representations” which are effector independent [14], [16], [18], [53], [54]. Consequently, activity in left M1_ipsi_ might reflect input deriving from the left parietal-premotor networks, that are involved in controlling complex movements even when these are executed with the ipsilateral body side. This view is supported by several studies showing that disrupting M1 activity by repetitive TMS leads to performance decrements when the ipsilateral hand executes a *complex* motor task [41], [55], [56]. In line with our results it has been shown that the disruptive effect during demanding motor control was stronger when left than when right M1_ipsi_ was disrupted [41]. An important area that can modulate activity in both hemispheres is the premotor cortex [57]. Premotor areas of the left hemisphere are specifically involved in the preparation and execution of motor actions of either hand [57], [58] whereas right premotor areas are important to prevent unwanted mirror activity in M1_ipsi_ [59]. Thus, hemispheric differences in the facilitation and %SICI of M1_ipsi_ are likely to reflect the differential involvement of the left versus right premotor cortex in complex motor control.

However, more research is needed to delineate the differential contributions of mechanisms acting at the level of spinal cord, transcallosal M1-M1 interaction or premotor-parietal circuits.
